# A Review of Keratin-Based Biomaterials for Biomedical Applications

**DOI:** 10.3390/ma3020999

**Published:** 2010-02-03

**Authors:** Jillian G. Rouse, Mark E. Van Dyke

**Affiliations:** Wake Forest Institute for Regenerative Medicine, Wake Forest University, Winston-Salem, North Carolina, USA; E-Mail: jrouse@wfubmc.edu (J.G.R.)

**Keywords:** keratin, human hair protein, natural biomaterial, protein film, scaffold

## Abstract

Advances in the extraction, purification, and characterization of keratin proteins from hair and wool fibers over the past century have led to the development of a keratin-based biomaterials platform. Like many naturally-derived biomolecules, keratins have intrinsic biological activity and biocompatibility. In addition, extracted keratins are capable of forming self-assembled structures that regulate cellular recognition and behavior. These qualities have led to the development of keratin biomaterials with applications in wound healing, drug delivery, tissue engineering, trauma and medical devices. This review discusses the history of keratin research and the advancement of keratin biomaterials for biomedical applications.

## 1. Introduction 

One of the primary goals of biomaterials research is the development of a matrix or scaffolding system that mimics the structure and function of native tissue. For this purpose, many researchers have explored the use of natural macromolecules due to their intrinsic ability to perform very specific biochemical, mechanical and structural roles. In particular, protein-based biomaterials have emerged as potential candidates for many biomedical and biotechnological applications due their ability to function as a synthetic extracellular matrix that facilitates cell-cell and cell-matrix interactions. Such substrates contain a defined, three-dimensional microstructure that supports cellular proliferation and cell-guided tissue formation, both of which are important characteristics for biomaterial scaffolds. In addition, the strong bioactivities and diverse physiochemical properties of proteinaceous macromolecules are attractive for other biomedical applications for which biocompatibility is essential, such as medical devices, bioactive surfaces, hygiene products, etc.

Several proteins have been investigated in the development of naturally-derived biomaterials, including collagen, albumin, gelatin, fibroin and keratin. Of these, keratin-based materials have shown promise for revolutionizing the biomaterial world due to their intrinsic biocompatibility, biodegradability, mechanical durability, and natural abundance. This review focuses on the history of keratin research and the development of keratin-based biomaterials for biomedical applications. A brief review of keratin biology is also discussed with an emphasis on how the proteins are developed within the hair fiber. 

## 2. Keratin Biology

The term “keratin” originally referred to the broad category of insoluble proteins that associate as intermediate filaments (IFs) and form the bulk of cytoplasmic epithelia and epidermal appendageal structures (*i.e.*, hair, wool, horns, hooves and nails). Subsequent research of these structural proteins led to the classification of mammalian keratins into two distinct groups based on their structure, function and regulation. “Hard” keratins form ordered arrays of IFs embedded in a matrix of cystine-rich proteins and contribute to the tough structure of epidermal appendages. “Soft” keratins preferentially form loosely-packed bundles of cytoplasmic IFs and endow mechanical resilience to epithelial cells [1,2,3]. In 2006, Schweizer *et al.* [4] developed a new consensus nomenclature for hard and soft keratins to accommodate the functional genes and pseudogenes for the full complement of human keratins. This system classifies the 54 functional keratin genes as either epithelial or hair keratins. The structural subunits of both epithelial and hair keratins are two chains of differing molecular weight and composition (designated types I and II) that each contain non-helical end-terminal domains and a highly-conserved, central alpha-helical domain. The type I (acidic) and type II (neutral-basic) keratin chains interact to form heterodimers, which in turn further polymerize to form 10-nm intermediate filaments. Although hard and soft keratins have closely related secondary structures, distinct differences in amino acid sequences contribute to measurable differences between the filamentous structures. Most notably, hair keratins contain a much higher content of cysteine residues in their non-helical domains and thus form tougher and more durable structures via intermolecular disulfide bond formation [2,5,6]. 

### 2.1. Hair Keratins

Hair fibers are elongated keratinized structures that are composed of heavily crosslinked hard keratins. Each fiber is divided into three principle compartments: the cuticle, cortex, and medulla. The thin outer surface of the fiber, the cuticle, is a scaly tubular layer that consists of over-lapping flattened cells. The cuticle primarily contains beta-keratins that function to protect the hair fiber from physical and chemical damage. The major body of the hair fiber is referred to as the cortex, which is composed of many spindle-shaped cells that contain keratin filaments. Occasionally, in the very center of the hair fiber is a region called the medulla that consists of a column of loosely connected keratinized cells [7]. 

Within the cortex of the hair fiber are two main groups of proteins: (1) low-sulfur, “alpha” keratins (MW 40−60 kDa) and (2) high-sulfur, matrix proteins (MW 10−25 kDa). Collectively, the hair fiber consists of 50−60% alpha keratins and 20−30% matrix proteins [7]. The alpha keratins assemble together to form microfibrous structures known as keratin intermediate filaments (KIFs) that impart toughness to the hair fiber. The matrix proteins function primarily as a disulfide crosslinker or glue that holds the cortical superstructure together and are also termed keratin associated proteins or KAPs [4]. In total, there are 17 human hair keratin genes (11 type I; 6 type II) [4] and more than 85 KAP genes [8] that potentially contribute to the hair structure in humans.

### 2.2. Development of Hair Keratins

Hair morphogenesis begins in a proliferative compartment at the base of the hair follicle called the bulb. Within this region, cells divide and differentiate to form the various compartments of the hair follicle. The hair follicle is a cyclic regeneration system comprised of actively migrating and differentiating stem cells responsible for the formation and growth of hair fibers. The follicle undergoes a continuous cycle of proliferation (anagen), regression (catagen), and quiescence (telogen) that is regulated by over thirty growth factors, cytokines and signaling molecules [9,10]. The mature anagen hair follicle contains a concentric series of cell sheaths, the outermost of which is called the outer root sheath (ORS), followed by a single cell layer called the companion sheath. The inner root sheath (IRS) lies adjacent to the companion layer and consists of three compartments: the Henle layer, the Huxley layer, and the IRS cuticle. The hair fiber fills the center of this multilayered cylinder, which is itself divided into cuticle, cortex and medulla [8,9,10]. As cells within the hair shaft terminally differentiate, they extrude their organelles and become tightly packed with keratin filaments. The cysteine-rich keratins become physically crosslinked upon exposure to oxygen and give strength and flexibility to the hair shaft [10].

Keratin genes have complex, differential, and in many cases sequential expression patterns within the cuticle and cortex of the hair follicle [5,11,12,13,14]. For example, only a few keratins are expressed in the hair-forming matrix of the cortex and cuticle, whereas others are sequentially switched on upon differentiation in the lower cortex. The bulk of keratins are expressed in the middle cortex (“keratinizing zone”) of the ascending hair fiber. Other keratin expressions are restricted to the hair cuticle and are sequentially expressed during hair morphogenesis [5,13]. The highly regulated expression pattern of keratins during hair morphogenesis is indicative of the functional differences between acidic and basic keratins, although this relationship is not yet fully understood [11,12]. 

## 3. History of Keratin Biomaterials

### 3.1. Early Uses of Keratins

The earliest documented use of keratins for medicinal applications comes from a Chinese herbalist named Li Shi-Zhen in the 16^th^ century. Over a 38-year period, Shi-Zhen wrote a collection of 800 books known as the *Ben Cao Gang Mu* that describe more than 11,000 therapeutic prescriptions. Among them is a substance made of ground ash from pyrolized human hair that was used to accelerate wound healing and blood clotting called Xue Yu Tan, also known as Crinis Carbonisatus. Although the details about the discovery of the biological activity of human hair are not reported in great detail, its uses for medicinal purposes are clearly documented [15].

The word “keratin” first appears in the literature around 1850 to describe the material that made up hard tissues such as animal horns and hooves (keratin comes from the Greek “*kera”* meaning horn). At the time, keratins intrigued scientists because they did not behave like other proteins. In particular, normal methods for dissolving proteins were ineffective for solubilizing keratin. Although methods such as burning and grinding had been known for some time, many scientists and inventors were more interested in dissolving hair and horns in order to make better products. The resolution to the insolubility problem came in 1905 with the issue of a United States patent to John Hoffmeier that described a process for extracting keratins from animal horns using lime. The extracted keratins were used to make keratin-based gels that could be strengthened by adding formaldehyde [16]. 

During the years from 1905 to 1935, many methods were developed to extract keratins using oxidative and reductive chemistries [17,18,19,20,21,22]. These technologies were initially applied to animal horns and hooves, but were also eventually used to extract keratins from wool and human hair. The biological properties of the extracts led to increased interest in the development of keratins for medical applications, and among the first inventions were keratin powders for cosmetics, composites, and coatings for drugs [23,24,25]. 

During the 1920s, keratin research changed its focus from products made from keratin to the structure and function of keratin proteins. Several key papers were published that analyzed oxidatively and reductively extracted keratins [21,22]. These scientists soon concluded that many different forms of keratin were present in these extracts, and that the hair fiber must be a complex structure, not simply a strand of protein. In 1934, a key research paper was published that described different types of keratins, distinguished primarily by having different molecular weights [22]. This seminal paper demonstrated that there were many different keratin homologs, and that each played a different role in the structure and function of the hair follicle.

### 3.2. Keratin Research from 1940−1970

It was during the years of World War II and immediately after that one of the most comprehensive research projects on the structure and chemistry of hair fibers was undertaken. Driven by the commercialization of synthetic fibers such as Nylon and polyester, Australian scientists were charged with protecting the country’s huge wool industry. Synthetic fibers were seen as a threat to Australia’s dominance in wool production, and the Council for Scientific and Industrial Research (later the Commonwealth Scientific and Industrial Research Organisation or CSIRO) established the Division of Protein Chemistry in 1940. The goal of this fundamental research was to better understand the structure and chemistry of fibers so that the potential applications of wool and keratins could be expanded. Earlier work at the University of Leeds and the Wool Industries Research Association in the UK had shown that wool and other fibers were made up of an outer cuticle and a central cortex. Building on this information, scientists at CSIRO conducted many of the most fundamental studies on the structure and composition of wool. Using X-ray diffraction and electron microscopy, combined with oxidative and reductive chemical methods, CSIRO produced the first complete diagram of a hair fiber [26]. 

CSIRO scientists also conducted extensive studies on the wool proteins themselves. Many methods for the extraction, separation, and identification of these keratins were developed. Other fundamental studies included wool surface chemistry, processing of products, fellmongering (harvesting of wool from sheep), felting, carbonising, surface treatments, flammability, denaturation, chemical modification, dyeing, photochemical degradation, and application of polymers to wool. This monumental effort was conducted over a period of more than 30 years and resulted in over 660 publications, 20 patents, and three books. In the meantime, the use of oxidative and reductive chemistry to extract keratins from hair fibers was being applied by other scientists across the world. In The Netherlands, researchers patented a method for making films and textile fibers from reductively extracted keratins from ground up hooves [27].

Probably nowhere in the world was keratin research more active than in Japan. Between the years of 1940 and 1970, applications for keratin-based inventions submitted to the Japanese patent office numbered more than 700. This was a renaissance in keratin research that was trending toward the fundamentals of materials science and biomaterials. Driven by the development of reliable methods to solubilize keratins, researchers were beginning to understand the many sub-classes of keratins, and their different properties [28,29,30,31,32]. In 1965, CSIRO scientist W. Gordon Crewther and his colleagues published the definitive text on the chemistry of keratins [7]. This chapter in *Advances in Protein Chemistry* contained references to more than 640 published studies on keratins. 

### 3.3. Keratin Research from 1970-Present

Advances in the extraction, purification and characterization of keratins, led to the exponential growth of keratin materials and their derivatives. In the 1970s, methods to form extracted keratins into powders, films, gels, coatings, fibers, and foams were developed and published by several research groups [33,34,35]. All of these methods made use of the oxidative and reductive chemistries developed decades earlier, or variations thereof. 

The prospect of using keratin as a biomaterial in medical applications was obvious. During the 1980s, collagen became a commonly used biomolecule in many medical applications. Other naturally derived molecules soon followed such as alginates from seaweed, chitosan from shrimp shells, and hyaluronic acid from animal tissues. The potential uses of keratins in similar applications began to be explored by a number of scientists. In 1982, Japanese scientist published the first study describing the use of a keratin coating on vascular grafts as a way to eliminate blood clotting [36], as well as experiments on the biocompatibility of keratins [37]. Soon thereafter in 1985, two researchers from the UK published a review article speculating on the prospect of using keratin as the building block for new biomaterials development [38]. In 1993, a Japanese scientists published a commentary on the prominent position keratins could take at the forefront of biomaterials development [39]. 

## 4. Keratin Biomaterials

The solid foundation for keratin research led to the development of many keratin-based biomaterials for use in biomedical applications. This foundation is based on several key properties of keratins that contribute to the overall physical, chemical and biological behavior of these biomaterials. First, extracted keratin proteins have an intrinsic ability to self-assemble and polymerize into porous, fibrous scaffolds. The spontaneous self-assembly of keratin solutions has been studied extensively at both the microscale [40,41,42] and macroscale levels [43]. This phenomenon of self-assembly is evident in the highly conserved superstructure of the hair fiber and, when processed correctly, is responsible for the reproducible architecture, dimensionality and porosity of keratin-based materials. In addition, keratin biomaterials derived from wool and human hair have been shown to possess cell binding motifs, such as leucine-aspartic acid-valine (LDV) and glutamic acid-aspartic acid-serine (EDS) binding residues, which are capable of supporting cellular attachment [44,45]. Together, these properties create a favorable three dimensional matrix that allows for cellular infiltration, attachment and proliferation. Like other intermediate filaments, keratins are also believed to participate in some regulatory functions that mediate cellular behavior [46,47]. Thus, the conservation of biological activity within regenerated keratin biomaterials could prove advantageous for the control of specific biological functions in a variety of tissue engineering applications. 

The enhanced physical, chemical and biological properties of keratins as well as the desire to exploit wool and human hair fibers as a renewable natural resource have fueled keratin biomaterials research over the past three decades. Much has been done to both fabricate and characterize new keratin-based products such as films, sponges, scaffolds and fibers. In many cases, these novel keratin materials have been shown to possess excellent biocompatibility. In addition, many researchers have discovered methods for modulating the physical and mechanical properties of keratins in order to create biomaterials that have appropriate characteristics for their application of interest. 

### 4.1. Keratin Films 

The preparation of protein films from keratin extracted from wool and human hair has been used for a number of years to explore the structural and biological properties of self-assembled keratins. Yamauchi *et al.* [48] were among the first to begin to investigate the properties of products made from extracted wool keratins and in doing so described the physiochemical and biodegradational properties of solvent-cast keratin films. Although pure keratin films were too fragile for practical use, the addition of glycerol resulted in a transparent, relatively strong, flexible, and biodegradable film [48]. In an additional publication, Yamauchi *et al.* [49] described the cell compatibility of this film by cultivation of mouse fibroblasts on the surface. When compared to the growth of cells on collagen and glass, the keratin substrate proved to be more adhesive to the cells and more supportive of cellular proliferation [49]. Fujii *et al.* [50] also demonstrated that hair keratins were useful for preparing protein films and described a rapid casting method. This research also revealed the feasibility of incorporating such bioactive molecules as alkaline phosphatase into the keratin films for controlled-release applications. The films, however, had poor strength and flexibility [50]. Together these early studies demonstrated the feasibility of preparing keratin films and demonstrated their potential for use as biomaterials in medical applications. 

Like many natural-derived biomaterials, however, the practical use of keratin-based products was ultimately limited by their poor mechanical characteristics. Thus, keratin film research shifted to focus on the optimization of the physical strength and flexibility of films while maintaining their excellent biological activity. Several approaches for controlling the physical and biological properties have been considered, including the addition of natural [51,52,53,54,55,56] and synthetic [57,58] polymers to keratin-blended systems and new preparation techniques for pure keratin films [59,60].

In 2002, Yamauchi’s group enhanced the mechanical properties of their glycerol containing keratin films by the addition of chitosan. Chitosan is a well investigated biomolecule for biomaterial applications, and is known to possess high biocompatibility and biological function for wound healing and antibacterial activity. Addition of chitosan into the keratin films resulted in improved mechanical strength. Furthermore, the chitosan-keratin films also demonstrated antibacterial properties and were shown to be good substrates for cell culture [51]. The biological activity of keratin films was also increased by incorporating a cell adhesion peptide, Arg-Gly-Asp-Ser (RGDS), at the free cysteine residues of reduced keratin extracts. RGDS-carrying keratin films proved to be excellent substrates for mammalian cell growth, and this work again demonstrated the potential and versatility of keratin biomaterials [52].

Silk fibroin (SF) is another natural polymer that has received much attention as a biomaterial due to its intrinsic biocompatibility and biodegradability. Keratin-SF films have been studied extensively in order to understand the interactions that occur between the two biomolecules and how they relate to the overall mechanical and biological characteristics of the biomaterial. Lee *et al.* [53] studied the secondary structure of keratin-SF films and observed a transition from random coil to β-sheet structure for fibroin due to the presence of the polar amino acids present in keratin. These blended films were shown to have enhanced antithrombogenicity properties and increased biocompatibility in comparison to SF or keratin only films [54], most likely due to the enhanced surface polarity of the blends generated by the conformational transformation of the proteins [55]. Vasconcelos *et al.* [56] further explored the mechanical and degradation properties of keratin-SF blended films and concluded that SF and keratin interactions are not simply additive but rather the two proteins are capable of unique intermolecular interactions that directly affect the bulk properties of the films. Ultimately, the nature and strength of these interactions and knowledge of the degradation rates will allow for the design of matrices for release of active compounds that are suitable for future biomedical applications [56]. 

In addition to natural biopolymers, the interaction between keratin and synthetic polymers has also been studied [57,58]. Tonin *et al.* [57] explored the relationship between poly(ethylene oxide) (PEO) and keratin blended films in order to develop a keratin-based material with improved structural properties. Morphological, structural and thermal analyses of the keratin/PEO films revealed that at appropriate levels, keratin inhibits PEO crystallization and PEO interferes with the keratin self-assembly, inducing a more thermally-stable, β-sheet secondary protein structure. The improved structural properties of keratin/PEO blends enables the development of keratin materials for use as scaffolds for cell growth, wound dressings and drug delivery membranes [57]. The intermolecular interactions between keratin and polyamide 6 (PA6) have also been studied with the goal of creating keratin-based materials that have practical use for a wide variety of applications ranging from biomedical devices to active water filtration and textile fibers [58]. 

In addition to creating blended keratin systems with natural or synthetic polymers, researchers have also investigated alternative fabrication techniques for creating keratin films with more suitable mechanical properties. Katoh *et al.* [60] reported an alternative method for processing keratin films to overcome the limited versatility associated with solution-cast methods. Compression molding of S-sulfo keratin powder proved to be an effective technique for producing pure keratin films of distinct shape. Control of the mechanical properties of the films was obtained by controlling the molding temperature and water content of the film, and the biocompatibility of the S-sulfo films was also demonstrated by fibroblast attachment and proliferation on the keratin substrates [60]. In a separate study, an improved procedure for preparing pure keratin films with translucent and flexible properties was reported, and the practical application and compatibility of the films were demonstrated by testing their compatibility with human skin [59]. 

Recently, Reichl *et al.* [61] characterized two different approaches for substrate coatings and demonstrated the growth behavior of twelve different cell lines cultured on the keratin films. Results showed that growth substrates formed by casting of a keratin nanosuspension supported cell adherence and improved cell growth as compared to uncoated polystyrene or keratin coatings formed by trichloroacetic acid precipitation. The new approach is believed to be a low cost, standardized alternative to commonly used coatings such as collagen and fibronectin [61]. 

### 4.2. Keratin Sponges and Scaffolds

The ability of extracted keratin proteins to self-assemble and polymerize into complex three dimensional structures has led to their development as scaffolds for tissue engineering. Fabrication of wool keratin scaffolds for long term cell cultivation was first reported by Tachibana *et al.* [44] in 2001. The matrices were created by lyophilization of aqueous wool keratin solutions after controlled freezing, which resulted in a rigid and heat-stable structure with a homogenously porous micro-architecture. The keratins, which were shown to contain RGD and LDV cell adhesion sequences, exhibited good cell compatibility and supported the attachment and proliferation of fibroblasts over a long-term cultivation period of 23−43 days. In addition, the free cysteine residues present within the scaffold were shown to be potential modification sites for the immobilization of bioactive substances [44]. In later work, lysozyme was used as a model compound and linked to the keratin sponge via disulfide and thioether bonds. Disulfide-linked lysozyme was gradually released over a 21-day period whereas lysozyme linked via thioether bonds was stably maintained for up to two months. This work demonstrated that the selection of a chemical crosslinker can uniquely determine the stability of an immobilized bioactive substance on keratin sponges [62]. 

Functionalization of active free thiol in the keratin sponges using various chemical treatments has also been demonstrated using iodoacetic acid, 2-bromoethylamine, and iodoacetamide to produce carboxyl-, amino-, and amido-sponges, respectively. These chemically-modified keratin sponges have been shown to mimic extracellular matrix proteins, and the large presence of active groups within the sponges has allowed for further hybridization with bioactive molecules. This technique was demonstrated by Tachibana *et al.* [63] in 2005 with the hybridization of keratin sponges with calcium phosphate. Two types of calcium phosphate composite sponges were fabricated by either chemically binding calcium and phosphate ions or trapping hydroxyapatite particles within the keratin carboxy-sponges. Both hybridized materials supported osteoblast cultivation and altered their differentiation pattern based on the expression pattern of alkaline phosphatase [63]. Keratin carboxy-sponges have also been functionalized with bone morphogenetic protein-2 (BMP-2), which was shown to associate tightly within the keratin sponge and to localize the differentiation of preosteoblasts grown with the construct. Cells outside of the BMP-2-loaded construct did not differentiate, suggesting that no significant amount of BMP-2 leaked out and that the effects were confined inside the modified keratin sponge. These findings are significant for *in vivo* applications because it is expected that the use of these scaffolds will promote internal osteogenesis while avoiding external heterotopic ossification [64].

Regulation of pore size and porosity of keratin scaffolds was achieved by Katoh *et al.* [65] using a compression molding/particulate leaching (CM/PL) technique. The ability to regulate the pore diameter and interconnectivity of scaffolds for tissue engineering applications is desired for allowing adequate cellular infiltration and nutrient delivery. In addition to having regulated pore size, scaffolds created using the CM/PL method were water tolerable, which presents significant superiority over collagen materials that are soluble in water without the use of UV irradiation or cytotoxic chemical crosslinkers [65].

The *in vivo* biodegradation of keratin bars was explored by Peplow *et al.* [66] in order to establish a relationship between mass and physical strength. Rectangular bars of reconstituted keratins were subcutaneously implanted into adult rats, and dry weight and elastic modulus of the explanted bars were monitored over an 18-week time period. The dry weight of the bars decreased gradually with a maximum weight degradation of 22% at 18 weeks. The elastic modulus of the keratin bars decreased abruptly between 3 and 6 weeks accompanied by an increase in the number of fissures and cavitations at the surface of the bars. This gradual degradation and quick loss of mechanical integrity are indications that this form of keratin is more suited as a resorbable implant material to provide scaffolding for non-load bearing applications [66].

The construction, characterization and cytocompatibility of human hair protein scaffolds for *in vitro* tissue engineering applications has recently been reported by Verma *et al.* [45]. Keratin proteins extracted from hair were fabricated into porous sponges via lyophilization of frozen protein suspensions. Characterization of the sponges was performed using swelling experiments and morphological assessments made by scanning electron microscopy (SEM), which showed that the sponges were capable of swelling 48% within a period of 60 minutes and that the sponge surface had an average pore diameter of 150 µm. The interconnectivity and pore diameters supported cell attachment and survival. The authors suggest that these scaffolds are prospective materials for tissue engineering applications due to their human origin, biodegradability and cytocompatibility [45]. 

### 4.3. Keratin Fibers

In recent years, research on the electrospinning of biocompatible polymeric materials has greatly increased due to the abundance of potential biomedical applications for nanofibrous materials. Electrospinning is a technique that utilizes a high voltage to create an electrically charged jet of polymer that is drawn toward a grounded collection plate or mandrel. The resulting fibers have diameters in the nano- to micro-scale range and are randomly arranged to form a non-woven fibrous mat. The enhanced physical configuration (*i.e.*, small pore size, high porosity, three-dimensional features, and high surface area-to-volume ratio) of nanostructured nonwovens promotes cell adhesion and growth, which has led to the development of electrospun membranes for such uses as bandages for wound healing and scaffolds for tissue engineering. Recently, the electrospinning process has also been extended to include regenerated keratin extracted from hair and wool fibers. Due to the intrinsically poor mechanical characteristics of pure keratin, however, many researchers have resorted to the addition of synthetic or natural polymers in order to increase the processability of keratin for fiber formation. Much work has been done to characterize the intermolecular interactions between the keratin and “additive” macromolecule in order to correlate the properties of the blend solution to the properties of the electrospun fibers. 

Aluigi *et al.* [67,68] created keratin/PEO materials by combining aqueous keratin solutions and PEO powder. In the first of two studies, the investigators identified the electrospinning parameters to create defect-free fibrous materials. Blended solutions with a keratin/PEO weight ratio of 50:50 and 7% and 10% total polymer concentrations were shown to have sufficient viscosities to electrospin with few defects. Spectroscopic and thermal analyses indicated that the electrospinning process destabilized the natural self-assembly of keratin and promoted a less complex protein conformation [67]. In further work, keratin and PEO were combined in different proportions in order to correlate the chemical, physical, and rheological properties of the blend solutions with the morphological, structural, thermal and mechanical properties of the electrospun mats. The keratin/PEO solutions were shown to have increased viscosities in comparison to both pure PEO and keratin, and the blends exhibited a non-Newtonian flow behavior with strong shear-thinning properties that were dependent on PEO concentration. The low viscosity of blends with higher keratin content greatly hindered their ability to form fibers; however, solutions with a lower composition of keratin were successfully electrospun without defects. Comparisons between actual and theoretical rheological properties using Graessley’s theory showed that the broadening of molecular weight distribution and possible bonding between PEO and keratin macromolecules at certain keratin/PEO ratios are responsible for the shear viscosity behavior of the blends, which ultimately correlate with the morphology of the electrospun fibers [69]. The practical uses of the keratin/PEO nanofibrous mats, however, were ultimately limited by their water instability and poor mechanical properties [68].

Fibroin regenerated from silk has also been used to improve the processability of keratin for electrospinning applications [70]. Characterizations of the rheological behavior of keratin/fibroin solutions revealed macromolecule interactions that promoted the formation of network structures with maximum synergy at a 50/50 (w/w) blend ratio. At this ratio, the synergistic effects on the protein interactions resulted in the formation of smaller-diameter, finer nanofibers as compared to fibers formed using solutions of unequal ratios of keratin/fibroin. Conformational analyses confirmed the prevalence of β-sheet secondary structure in keratin/fibroin films except at the 50/50 blend in which the proteins showed a propensity to assemble in the α-helix-coiled structure. On the contrary, the electrospinning process was shown to induce changes in secondary structure at all blend ratios by preventing β-sheet formation and promoting a random coil or α-form structure. In addition, the α-crystallites formed by electrospinning were shown to be less thermally stable, most likely due to the high rate of fiber formation that limits the molecular rearrangement and crystallization of the keratin chains [70].

Wet-spinning is another fiber-forming technique that has traditionally been used for manufacturing synthetic fibers for the textile industry, but has recently been employed to create single fiber biomaterials. This method involves extrusion of a dope solution through a spinneret into a coagulation bath and subsequent drawing/stretching to promote polymer chain alignment and fiber formation. The physical limitations of keratin materials have hindered the production of pure keratin fibers, yet researchers have overcome these challenges using blends of synthetic and natural polymers with improved material properties. 

Katoh *et al.* [71] improved upon the fiber-forming capabilities of aqueous keratin solutions using poly-(vinyl alcohol) (PVA). PVA acted to increase the viscosity of the spinning dope, which allowed fibers with a keratin content ranging from 13−46% to be spun. Due to the fragility of fibers with high amounts of keratin, the maximum keratin content for sufficient fiber formation was determined to be 30%. This combination of keratin and PVA proved to be advantageous in terms of mechanical strength, waterproof characteristics, and the adsorption of toxic substances. According to the authors, keratin-PVA fibers are expected to have wide-spread industrial applications as absorbents for toxic substances such as heavy metals ions and formaldehyde gas [71]. 

Wrzesniewska-Toski *et al.* [72] also employed wet-spinning techniques to create novel fibrous keratin-based materials that have potential application as hygienic fabrics. Keratin extracted from chicken feathers and bio-modified cellulose were combined and used to create fibers that were characterized as having better sorption properties, higher hygroscopicity, and a smaller wetting angle than cellulose-only fibers. Although introduction of keratin into cellulose fibers decreased the mechanical properties, a level was achieved that still enabled their application for manufacturing composite fibrous materials. In addition, the cellulose-keratin fibers had better biodegradation than cellulose fibers. 

## 5. Keratin Biomaterials in Tissue Engineering and Regenerative Medicine

Much work has been done to fabricate and characterize keratin-based materials and to demonstrate their cytocompatibility and biodegradation. Until recently, however, few of these biomaterial developments had been applied in models of tissue regeneration. 

Sierpinski *et al.* [73] and Apel *et al.* [74] demonstrated that keratin-based hydrogels were neuroinductive and capable of facilitating regeneration in a peripheral nerve injury model in mice. Human hair keratins enhanced the *in vitro* activity of Schwann cells by inducing cellular proliferation and migration, and by upregulating expression of specific genes required for important neuronal functions. When translated into a mouse tibial nerve injury model, keratin gel-filled conduits served as a neuroinductive provisional matrix that mediated axon regeneration and improved functional recovery compared to sensory nerve autografts [73]. In another study, the time course of peripheral nerve regeneration was evaluated with respect to neuromuscular recovery and nerve histomorphometry. Keratin-filled hydrogels were shown to accelerate nerve regeneration as evidenced by improved electrophysiological recovery and increased axon density at early time points. This early development of neuromuscular contacts resulted in more functional connections with the target muscle that in turn promoted increased axon myelination at six months. The authors concluded that these results showed that keratin-based scaffolds made from human hair can facilitate peripheral nerve regeneration and promote neuromuscular recovery that is equivalent to the gold standard, sensory nerve autografts [74]. 

Keratin hydrogels derived from human hair have also been shown to act effectively as a hemostatic agent in a rabbit model of lethal liver injury. In comparison to other commonly used hemostats (QuickClot^®^ and HemCon^®^ bandage), the keratin hemostatic gel improved 24 hr survival and performed consistently as well, if not better than, conventional hemostats in terms of total blood loss and shock index. The keratin gel used in these experiments acted on the injury site by instigating thrombus formation and by forming a physical seal of the wound site that acted as a porous scaffold to allow for cellular infiltration and granulose tissue formation [75]. The ability for keratin-based biomaterials to be translated into the human clinical setting is dependent on further research to elucidate the mechanisms by which these materials regulate hemostasis and nerve regeneration.

## 6. Conclusions

It would appear that keratin biomaterials have been in the collective conscience of materials researchers for many decades, yet there are no keratin biomaterials currently in clinical use. This comprehensive review has shown an impressive level of activity, diversity, and ingenuity, albeit at a relatively low level compared to other mainstream biomaterials. Keratin biomaterials possess many distinct advantages over conventional biomolecules, including a unique chemistry afforded by their high sulfur content, remarkable biocompatibility, propensity for self-assembly, and intrinsic cellular recognition. As these properties become better understood, controlled and exploited, many biomedical applications of keratin biomaterials will make their way into clinical trials.

## References

[1] Moll R., Franke W.W., Schiller D.L., Geiger B., Krepler R. (1982). The catalog of human cytokeratins: Patterns of expression in normal epithelia, tumors and cultured cells. Cell.

[2] Fraser R.D., MacRae T.P., Parry D.A., Suzuki E. (1986). Intermediate filaments in alpha-keratins. Proc. Natl. Acad. Sci. USA.

[3] Coulombe P.A., Bousquet O., Ma L., Yamada S., Wirtz D. (2000). The 'ins' and 'outs' of intermediate filament organization. Tr. Cell Biol..

[4] Schweizer J., Bowden P.E., Coulombe P.A., Langbein L., Lane E.B., Magin T.M., Maltais L., Omary M.B., Parry D.A., Rogers M.A., Wright M.W. (2006). New consensus nomenclature for mammalian keratins. J. Cell Biol..

[5] Moll R., Divo M., Langbein L. (2008). The human keratins: Biology and pathology. Histochem. Cell Biol..

[6] Yu J., Yu D., Checkla D.M., Freedberg I.M., Bertolino A.P. (1993). Human Hair Keratins. J. Invest. Dermatol..

[7] Crewther W.G., Fraser R.D.B., Lennox F.G., Lindley H., Anfinsen C.B., Anson M.L., Edsall J.T., Richards F.M. (1965). The Chemistry of Keratins. Advances in Protein Chemistry.

[8] Rogers M.A., Langbein L., Praetzel-Wunder S., Winter H., Schweizer J. (2006). Human hair keratin-associated proteins (KAPs). Int. Rev. Cytol..

[9] Stenn K.S., Paus R. (1999). What controls hair follicle cycling?. Exp. Dermatol..

[10] Alonso L., Fuchs E. (2006). The hair cycle. J. Cell Sci..

[11] Langbein L., Rogers M.A., Winter H., Praetzel S., Beckhaus U., Rackwitz H.R., Schweizer J. (1999). The catalog of human hair keratins. I. Expression of the nine type I members in the hair follicle. J. Biol. Chem..

[12] Langbein L., Rogers M.A., Winter H., Praetzel S., Schweizer J. (2001). The catalog of human hair keratins. II. Expression of the six type II members in the hair follicle and the combined catalog of human type I and II keratins. J. Biol. Chem..

[13] Langbein L., Schweizer J. (2005). Keratins of the human hair follicle. Int. Rev. Cytol..

[14] Schweizer J., Langbein L., Rogers M.A., Winter H. (2007). Hair follicle-specific keratins and their diseases. Exp. Cell Res..

[15] Zhen L.S. (2005). Ben Cao Gang Mu.

[16] Hofmeier J. (1905). Horn-lime plastic masses from keratin substances. German Pat..

[17] Breinl F., Baudisch O. (1907). The oxidative breaking up of keratin through treatment with hydrogen peroxide. Z Physiol. Chem..

[18] Neuberg C. (1909). Process of producing digestable substances from keratin. US Pat..

[19] Lissizin T. (1915). Behavior of keratin sulfur and cystin sulfur in the oxidation of these proteins by potassium permanganate I. Biochem. Bull..

[20] Zdenko S. (1924). Solubility and digestibility of the degradation products of albumoids I. Z Physiol. Chem..

[21] Lissizin T. (1928). The oxidation products of keratin by oxidation with permanganate II. Z Physiol. Chem..

[22] Goddard D.R., Michaelis L. (1935). Derivatives of Keratin. J. Biol. Chem..

[23] Beyer C. (1907). The keratin or horny substance of the hair. German Pat..

[24] Goldsmith B.B. (1909). Thermoplastic composition containing keratin. US Pat..

[25] Dale H.N. (1932). Keratin and other coatings for pills. Pharm. J..

[26] Rivett D.E., Ward S.W., Belkin L.M., Ramshaw J.A.M., Wilshire J.F.K. (1996). Keratin and Wool Research. The Lennox Legacy.

[27] van den Bergh J., Milo G.J., van Dijk H.E.P. (1941). Keratin-resin threads, films, *etc*. Netherlands Pat..

[28] Orwin D.F.G, Baumann H., Asquith R.S., Parry D.A.D., Parry D.A.D., Creamer L.K. (1979). Fibrous Proteins: Scientific, Industrial and Medical Aspects.

[29] Earland C., Knight C.S. (1956). Structure of keratin II: Amino acid content of fractions isolated from oxidized wool. Biochem. Biophys. Acta.

[30] Kikkawa M., Chonan Y., Toyoda H. (1974). Solubilization of keratin 6: Solubilization of feather keratin by oxidation with performic acid. Hikaku Kagaku.

[31] Buchanan J.H. (1977). A cystine-rich protein fraction from oxidized alpha-keratin. Biochem. J..

[32] Matsunga A., Chonan Y., Toyoda H. (1981). Studies on the chemical properties of human hair keratin, Part 1: Fractionation and amino acid composition of human hair solubilized by performic acid oxidation. Hikaku Kagaku.

[33] Anker C.A. (1972). Method of preparing keratin-containing films and coatings. US Pat..

[34] Kawano Y., Okamoto S. (1975). Film and gels of keratin. Kagaku Seibutsu.

[35] Okamoto S. (1977). Formation of films from some proteins. Nippon Shokuhin Kogyo Gakkaishi.

[36] Noishiki Y., Ito H., Miyamoto T., Inagaki H. (1982). Application of Denatured Wool Keratin Derivatives to an Antithrombogenic Biomaterial-Vascular Graft Coated with a Heparinized Keratin Derivative. Kobunshi Ronbunshu.

[37] Ito H., Miyamoto T., Inagaki H., Noishiki Y. (1982). Biocompatibility of Denatured Keratins from Wool. Kobunshi Ronbunshu.

[38] Jarman T., Light J. (1985). Prospects for novel biomaterials development. World Biotech Report.

[39] (1993). Various Authors Biomaterial forefront: Keratin which can be extracted by simple chemical technique. Kogyo Zairyo.

[40] van de Locht M. (1987). Reconstitution of microfibrils from wool and filaments from epidermis proteins. Melliand Textilberichte.

[41] Steinert P.M., Gullino M.I. (1976). Bovine epidermal keratin filament assembly *in vitor*. Biochem. Biophys. Res. Commun..

[42] Thomas H., Conrads A., Phan P.H., van de Locht M., Zahn H. (1986). *In vitor* reconstitution of wool intermediate filaments. Int. J. Biol. Macromol..

[43] Ikkai F., Naito S. (2002). Dynamic light scattering and circular dichroism studies on heat-induced gelation of hard-keratin protein aqueous solutions. Biomacromolecules.

[44] Tachibana A., Furuta Y., Takeshima H., Tanabe T., Yamauchi K. (2002). Fabrication of wool keratin sponge scaffolds for long-term cell cultivation. J. Biotechnol..

[45] Verma V., Verma P., Ray P., Ray A.R. (2008). Preparation of scaffolds from human hair proteins for tissue-engineering applications. Biomed. Mater..

[46] Magin T.M., Vijayaraj P., Leube R.E. (2007). Structural and regulatory functions of keratins. Exp. Cell Res..

[47] Izawa I., Inagaki M. (2006). Regulatory mechanisms and functions of intermediate filaments: A study using site- and phosphorylation state-specific antibodies. Cancer Sci..

[48] Yamauchi K., Yamauchi A., Kusunoki T., Kohda A., Konishi Y. (1996). Preparation of stable aqueous solution of keratins, and physiochemical and biodegradational properties of films. J. Biomed. Mater. Res..

[49] Yamauchi K., Maniwa M., Mori T. (1998). Cultivation of fibroblast cells on keratin-coated substrata. J. Biomat. Sci.-Polym. E..

[50] Fujii T., Ogiwara D., Arimoto M. (2004). Convenient procedures for human hair protein films and properties of alkaline phosphatase incorporated in the film. Biol. Pharm. Bull..

[51] Tanabe T., Okitsu N., Tachibana A., Yamauchi K. (2002). Preparation and characterization of keratin-chitosan composite film. Biomaterials.

[52] Yamauchi K., Hojo H., Yamamoto Y., Tanabe T. (2003). Enhanced cell adhesion on RGDS-carrying keratin film. Mat. Sci. Eng. C-Bio. S..

[53] Lee K.Y., Ha W.S. (1999). DSC studies on bound water in silk fibroin/S-carboxymethyl kerateine blend films. Polymer.

[54] Lee K.Y., Kong S.J., Park W.H., Ha W.S., Kwon I.C. (1998). Effect of surface properties on the antithrombogenicity of silk fibroin/S-carboxymethyl kerateine blend films. J. Biomater. Sci. Polym. Ed..

[55] Lee K.Y. (2001). Characterization of Silk/Fibroin/S-carboxymethyl Kerateine Surfaces: Evaluation of Biocompatibility by Contact Angle Measurements. Fibers Polym..

[56] Vasconcelos A., Freddi G., Cavaco-Paulo A. (2008). Biodegradable materials based on silk fibroin and keratin. Biomacromolecules.

[57] Tonin C., Aluigi A., Vineis C., Varesano A., Montarsolo A., Ferrero F. (2007). Thermal and structural characterization of poly(ethylene-oxide)/keratin blend films. J. Therm. Anal. Calorim..

[58] Zoccola M., Montarsolo A., Aluigi A., Varesano A., Vineis C., Tonin C. (2007). Electrospinning of polyamide 6/modified-keratin blends. E-Polym..

[59] Fujii T., Ide Y. (2004). Preparation of translucent and flexible human hair protein films and their properties. Biol. Pharm. Bull..

[60] Katoh K., Shibayama M., Tanabe T., Yamauchi K. (2004). Preparation and physicochemical properties of compression-molded keratin films. Biomaterials.

[61] Reichl S. (2009). Films based on human hair keratin as substrates for cell culture and tissue engineering. Biomaterials.

[62] Kurimoto A., Tanabe T., Tachibana A., Yamauchi K. (2003). Keratin sponge: Immobilization of lysozyme. J. Biosci. Bioeng..

[63] Tachibana A., Kaneko S., Tanabe T., Yamauchi K. (2005). Rapid fabrication of keratin-hydroxyapatite hybrid sponges toward osteoblast cultivation and differentiation. Biomaterials.

[64] Tachibana A., Nishikawa Y., Nishino M., Kaneko S., Tanabe T., Yamauchi K. (2006). Modified keratin sponge: Binding of bone morphogenetic protein-2 and osteoblast differentiation. J. Biosci. Bioeng..

[65] Katoh K., Tanabe T., Yamauchi K. (2004). Novel approach to fabricate keratin sponge scaffolds with controlled pore size and porosity. Biomaterials.

[66] Peplow P.V., Dias G.J. (2004). A study of the relationship between mass and physical strength of keratin bars *in vivo*. J. Mater. Sci. Mater. Med..

[67] Aluigi A., Varesano A., Montarsolo A., Vineis C., Ferrero F., Mazzuchetti G., Tonin C. (2007). Electrospinning of keratin/poly(ethylene oxide) blend nanofibers. J. Appl. Polym. Sci..

[68] Aluigi A., Vineis C., Varesano A., Mazzuchetti G., Ferrero F., Tonin C. (2008). Structure and properties of keratin/PEO blend nanofibres. Eur. Polym. J..

[69] Varesano A., Aluigi A., Vineis C., Tonin C. (2008). Study on the shear viscosity behavior of keratin/PEO blends for nanofibre electrospinning. J. Polym. Sci. Poly. Phys..

[70] Zoccola M., Aluigi A., Vineis C., Tonin C., Ferrero F., Piacentino M.G. (2008). Study on cast membranes and electrospun nanofibers made from keratin/fibroin blends. Biomacromolecules.

[71] Katoh K., Shibayama M., Tanabe T., Yamauchi K. (2004). Preparation and properties of keratin-poly(vinyl alcohol) blend fiber. J. Appl. Polym. Sci..

[72] Wrześniewska-Tosik K., Wawro D., Ratajska M., Stęplewski W. (2007). Novel composites with feather keratin. Fibres Text. East. Eur..

[73] Sierpinski P., Garrett J., Ma J., Apel P., Klorig D., Smith T., Koman L.A., Atala A., Van Dyke M. (2008). The use of keratin biomaterials derived from human hair for the promotion of rapid regeneration of peripheral nerves. Biomaterials.

[74] Apel P.J., Garrett J.P., Sierpinski P., Ma J., Atala A., Smith T.L., Koman L.A., Van Dyke M.E. (2008). Peripheral nerve regeneration using a keratin-based scaffold: Long-term functional and histological outcomes in a mouse model. J. Hand Surg. Am..

[75] Aboushwareb T., Eberli D., Ward C., Broda C., Holcomb J., Atala A., Van Dyke M. (2009). A Keratin biomaterial gel hemostat derived from human hair: Evaluation in a rabbit model of lethal liver injury. J. Biomed. Mater. Res. B.

